# Different cellulosic polymers for synthesizing silver nanoparticles with antioxidant and antibacterial activities

**DOI:** 10.1038/s41598-020-79834-6

**Published:** 2021-01-08

**Authors:** Ahmed A. H. Abdellatif, Hamad N. H. Alturki, Hesham M. Tawfeek

**Affiliations:** 1 Department of Pharmaceutics, College of Pharmacy, Qassim University, Buraidah, 51452 Kingdom of Saudi Arabia; 2 Department of Pharmaceutics and Industrial Pharmacy, Faculty of Pharmacy, Al-Azhar University, Assiut, 71524 Egypt; 3 Department of Industrial Pharmacy, Faculty of Pharmacy, Assiut University, Assiut, 71526 Egypt

**Keywords:** Nanobiotechnology, Drug discovery, Microbiology, Nanoscience and technology

## Abstract

The use of cellulosic polymers as efficient reducing, coating agents, and stabilizers in the formulation of silver nanoparticles (AgNPs) with antioxidant and antibacterial activity was investigated. AgNPs were synthesized using different cellulosic polymers, polyethylene glycol, and without polymers using tri-sodium citrate, for comparison. The yield, morphology, size, charge, in vitro release of silver ion, and physical stability of the resulting AgNPs were evaluated. Their antioxidant activity was measured as a scavenging percentage compared with ascorbic acid, while their antibacterial activity was evaluated against different strains of bacteria. The amount of AgNPs inside bacterial cells was quantified using an ICP-OES spectrometer, and morphological examination of the bacteria was performed after AgNPs internalization. Cellulosic polymers generated physically stable AgNPs without any aggregation, which remained physically stable for 3 months at 25.0 ± 0.5 and 4.0 ± 0.5 °C. AgNPs formulated using ethylcellulose (EC) and hydroxypropyl methylcellulose (HPMC) had significant (*p* ≤ 0.05; ANOVA/Tukey) antibacterial activities and lower values of MIC compared to methylcellulose (MC), PEG, and AgNPs without a polymeric stabilizer. Significantly (*p* ≤ 0.05; ANOVA/Tukey) more AgNPs-EC and AgNPs-HPMC were internalized in *Escherichia coli* cells compared to other formulations. Thus, cellulosic polymers show promise as polymers for the formulation of AgNPs with antioxidant and antibacterial activities.

## Introduction

Nanoparticles are now becoming a promising future for many researchers due to the numerous applications in biomedical devices, pharmaceuticals, and food industries. As well as, their ability to deliver drugs to specific tissues (e.g. anticancer drugs) and to enhance the tumor-killing effects of chemotherapeutic agents, in addition to their uses in cosmetic, household, and other health-related products^1^. In particular, silver nanoparticles (AgNPs) are widely used in different research areas due to their specific physical and chemical properties, which allow them to exert various activities, particularly in biomedical applications. AgNPs show antibacterial, antiviral, antifungal, antiangiogenic, antioxidant, antitumor, and anti-inflammatory activities, and can be used as drug carriers, imaging, water treatment, and biosensing materials^2^. In addition, AgNPs have clinical value in the treatment of infections and burns^3^. Khatoon et al*.*^4^ prepared AgNPs via reduction with tri-sodium citrate and found they significantly inhibited several Gram-positive (*Bacillus subtilis* and *Staphylococcus aureus*) and Gram-negative (*Escherichia coli*) bacteria. However, the toxicity of AgNPs in vitro and in vivo represent a great challenge in the synthesis and applications of AgNPs^5^. Thus, further research is needed to develop an optimal method for their synthesis.

AgNPs can be synthesized using several methods, including physical, chemical, and biological methods^6^. However, some methods, such as physical methods, produce a lower yield, as well as having an increased risk of solvent contamination. So far, biological methods are more environmentally friendly and can produce AgNPs with a uniform size distribution^6^. However, they are affected by several factors, including solvents, reducing agents, and toxic-free materials, and an additional step is required for the prevention of particle aggregation^7^. Chemical methods are the most commonly used for the production of AgNPs because of their high yield, simplicity, and low cost. However, the choice of reducing agent is key for the successful production of stable AgNPs with low cytotoxicity, since most reducing agents are toxic compounds^8,9^. In addition, each reducing agent has characteristic features that will affect the properties of the produced AgNPs. As a result, extensive research has been conducted for the development of other methods for synthesis of more safe non-toxic AgNPs with increased nanoparticle efficiency to allow for a greater control over particle size distribution, and morphology^10^.

Several factors could also affect the biological activity of chemically synthesized AgNPs (e.g. particles morphology and composition, coating or capping materials, ion release, size distribution, and type of reducing agent)^11^. Furthermore, the characterization of NPs is also a challenge because some measurement properties are a method related to the type and concentration of reducing agents^5,12^. The temperature of the reaction, may also contribute to the successful synthesis of AgNPs since their synthesis is dependent on the reduction reaction of AgNO_3_ solution. For this reason, the effects of different materials for the preparation of AgNPs have been investigated with the aim of obtaining safer NPs, in particular via a green chemistry approach^13,14^. Scientists are looking forward for much more environmentally and safe methods for AgNPs fabrication and so far, the green methods for their formulation has been adapted^15^.

Up to date and searching in literature, utilizing the cellulosic polymers for efficient stable AgNPs preparation with an antioxidant and antibacterial effect did not discussed deeply. In this study, we harnessed the dual action of cellulosic polymers as a reducing agent and coating agent to develop in a simple step of procedure AgNPs with an enhanced physical stability compared to AgNPs prepared using PEG and those prepared without a stabilizer using sodium citrate. Furthermore, the effect of these materials on the antioxidant and antibacterial activities of the produced AgNPs was evaluated. Here, AgNPs were synthesized using cellulosic polymers (e.g. ethylcellulose (EC), methylcellulose (MC), and hydroxypropyl methylcellulose (HPMC). In addition, AgNPs stabilized polyethylene glycol 6000 (AgNPs-PEG) were prepared via reduction using sodium borohydride. AgNPs with tri-sodium citrate (AgNPs-citrate) were also prepared for comparison. The yield percentage of the AgNPs produced was determined, and the efficiency of synthesis and polymer coating were verified via UV–VIS and FT-IR spectroscopy. The size, morphology, charge, in vitro silver ion release, and physical stability (3 months) of the AgNPs were evaluated. Antioxidant activity was determined using the 2,2-Diphenyl-1-picrylhydrazyl (DPPH) scavenging percentage method, while antimicrobial activity was evaluated in different Gram-positive and Gram-negative bacteria, with MIC values calculated for each formulation. In addition, the amount of AgNPs internalized into bacterial cells was quantified using ICP-OES (inductively coupled plasma optical emission spectrometer) and the morphology of the selected bacterial species after AgNPs uptake was examined using transmission electron microscopy (TEM).

## Results and discussion

### Synthesis of AgNPs

**Figure 1:**
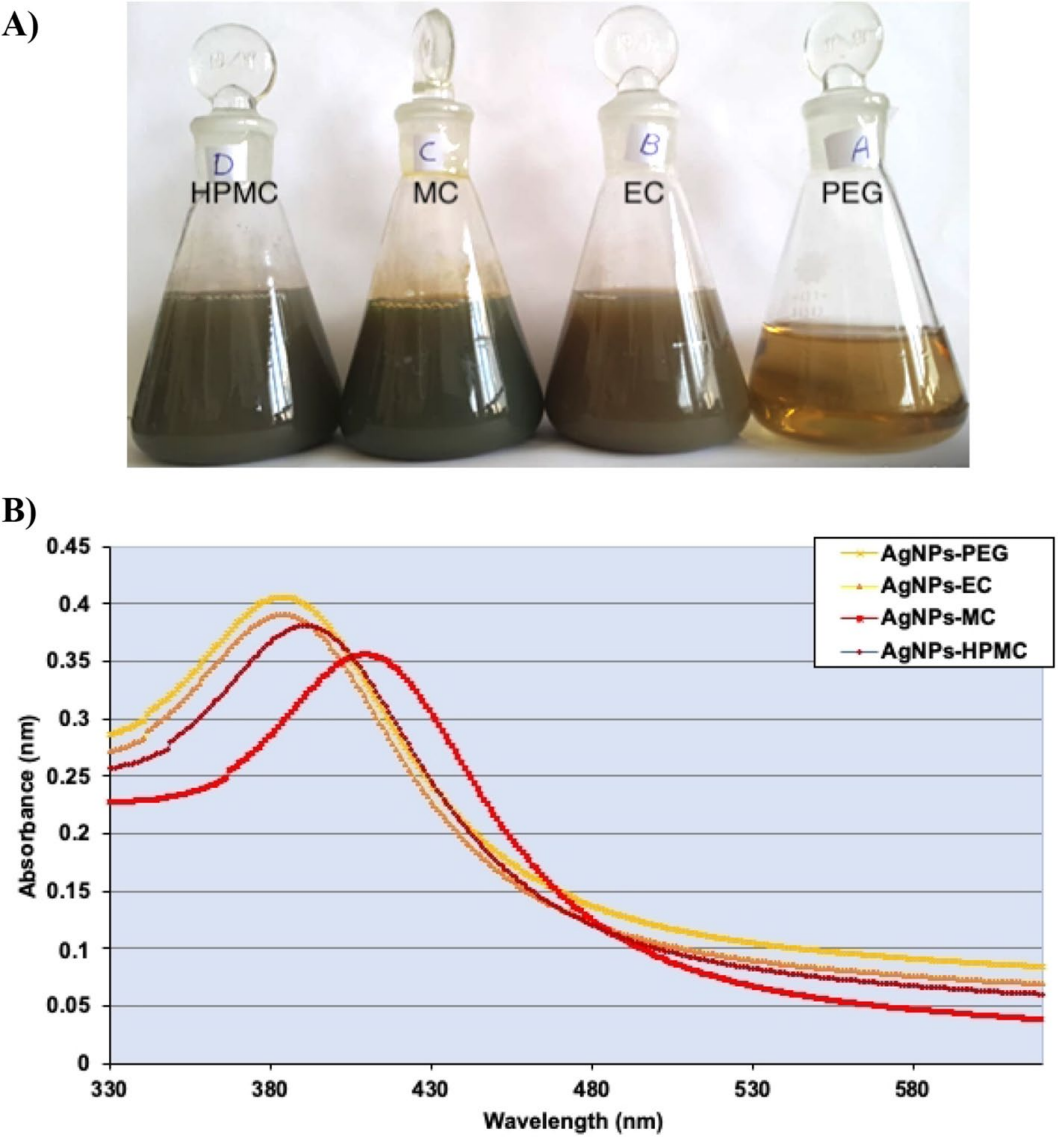
(**A**) Image of AgNPs solutions prepared using cellulosic polymers and PEG. (**B**) UV–VIS spectra of AgNPs prepared by PEG, EC, MC, and HPMC.

AgNPs with different colors were successfully obtained using various polymeric materials (Fig. 1A). The complete process of AgNO_3_ reduction was completed after 30 min of heating and boiling. AgNPs reduced with sodium borohydride and stabilized with PEG showed a characteristic yellow color. The complete reduction showed a change in color after ~ 18 min. AgNPs reduced with EC showed the start of a color change after ~ 16 min, and then turned dark brown after ~ 20 min, indicating complete reduction. AgNPs reduced with MC showed a color change after ~ 15 min, with a slight change in color. The color then became grayish-brown after ~ 30 min, indicating the complete reduction of the AgNO_3_ solution. Lastly, AgNPs reduced with HPMC showed a color change instantly, followed by the color became grayish-brown, stabilizing for 30 min of boiling of the solution. The results indicate that cellulosic materials may act as powerful reducing agents for the efficient preparation of AgNPs^16^.

AgNPs are synthesized by chemical reduction. However, certain chemical reducing agents are toxic and can harm humans after administration. Therefore, the use of reducing agents from natural resources for the preparation of AgNPs is highly recommended^17^. Cellulosic polymers are one of the richest resources in nature. Cellulosic derivatives can stabilize the formulated nanoparticles against aggregation^18^. Therefore, cellulosic derivatives act as reducing and stabilizing agents. Non-ionic substances were chosen as polymers for the synthesis of AgNPs, which have a positive adsorption at material interfaces. To synthesize AgNPs from the precursor AgNO_3_, polymers with reducing properties can be used, which also exert antioxidant activity, as will be shown^19^. Our results showed that the cellulosic polymers MC, EC, HPMC, and PEG exert reducing activity at comparatively different levels, confirming the applicability of these polymers as efficient reducing agents in the synthesis of AgNPs. The reducing power of the cellulosic polymers investigated, as well as PEG, was reflected by the values of the AgNPs yield percentage. MC has been reported to form a protective colloid that resists particle agglomeration^20^. Cellulosic AgNPs showed no aggregation or precipitation upon visual inspection after their synthesis, indicating that these cellulosic polymers function effectively as reducing and stabilizing agents, providing the resulting AgNPs with protection against aggregation. Moreover, these polymers have a negative charge that can react with Ag^+^ in the AgNO_3_ precursor, thus facilitating NP formulation and enhancing NP stability^18,21^.

### Characterization of AgNPs

#### Percentage yield of synthetized AgNPs

Table 1 displays the initial Ag^+^ and Ag^0^ concentrations in AgNPs after NPs synthesis. as determined using ICP-OES. Based on these concentrations, the yield percentage was determined. The concentrations of Ag^0^ in the AgNPs were 121.15 ± 12.7 µM (≈ 22.37%), 263.88 ± 8.05 µM (≈ 48.75%), 399.63 ± 4.3 µM (≈ 73.81%), 333.82 ± 9.03 µM (≈ 61.65%), and 124.57 ± 5.8 µM (≈ 23.1%) for AgNPs-PEG, AgNPs-EC, AgNPs-MC, AgNPs-HPMC, and AgNPs-CIT, respectively. Although the yield percentage did not reach 100%, an acceptably high yield percentage was obtained for both AgNPs-MC and AgNPs-HPMC, followed by AgNPs-EC. The inconsistencies between the measured Ag inputs and the theoretical inputs can be attributed to the adsorption of Ag^+^ or AgNPs by the centrifuge tubes or pipettes, volume loss, or transportation during sample transfer for dilution and estimation^22^.

**Table 1:** The yield percentage of Ag^0^ in AgNPs after conversion of Ag^+^ to Ag^0^ for the different prepared AgNPs formulations.

Sample	Conc. (µM)	Yield % (Conversion %)
AgNPs-PEG	121.1512.7	22.37 ± 2.5
AgNPs-EC	263.888.05	48.75 ± 3.1
AgNPs-MC	399.634.3	73.81 ± 4.3
AgNPs-HPMC	333.829.03	61.65 ± 2.9
AgNPs-CIT	124.575.8	23.1 ± 3.6
Ag^+^ (initial concentration)	541.49.5	‒

#### UV–VIS spectroscopy

The UV–VIS spectra indicated that AgNPs showed a strong absorption of electromagnetic waves in the visible region as a result of the surface plasmon resonance effect. Each peak has been smoothed for better visibility (Fig. 1B). The UV-spectroscopy recorded a maximum wavelength at 385, 381, 396, and 416.5 nm for AgNPs-PEG, AgNPs-EC, AgNPs-MC, and AgNPs-HPMC, respectively. These wavelengths were in agreement with those obtained by Hajji et al.^23^ indicating the successful preparation of AgNPs. In addition, the presence of one absorption peak for each preparation indicated the symmetrical geometry of the formulated AgNPs^24^. The shift observed in AgNPs-HPMC could be attributed to the difference in the shape and size distributions of the AgNPs, as previously reported^25^.

#### Fourier-transform infrared spectroscopy (FT-IR)

**Figure 2:**
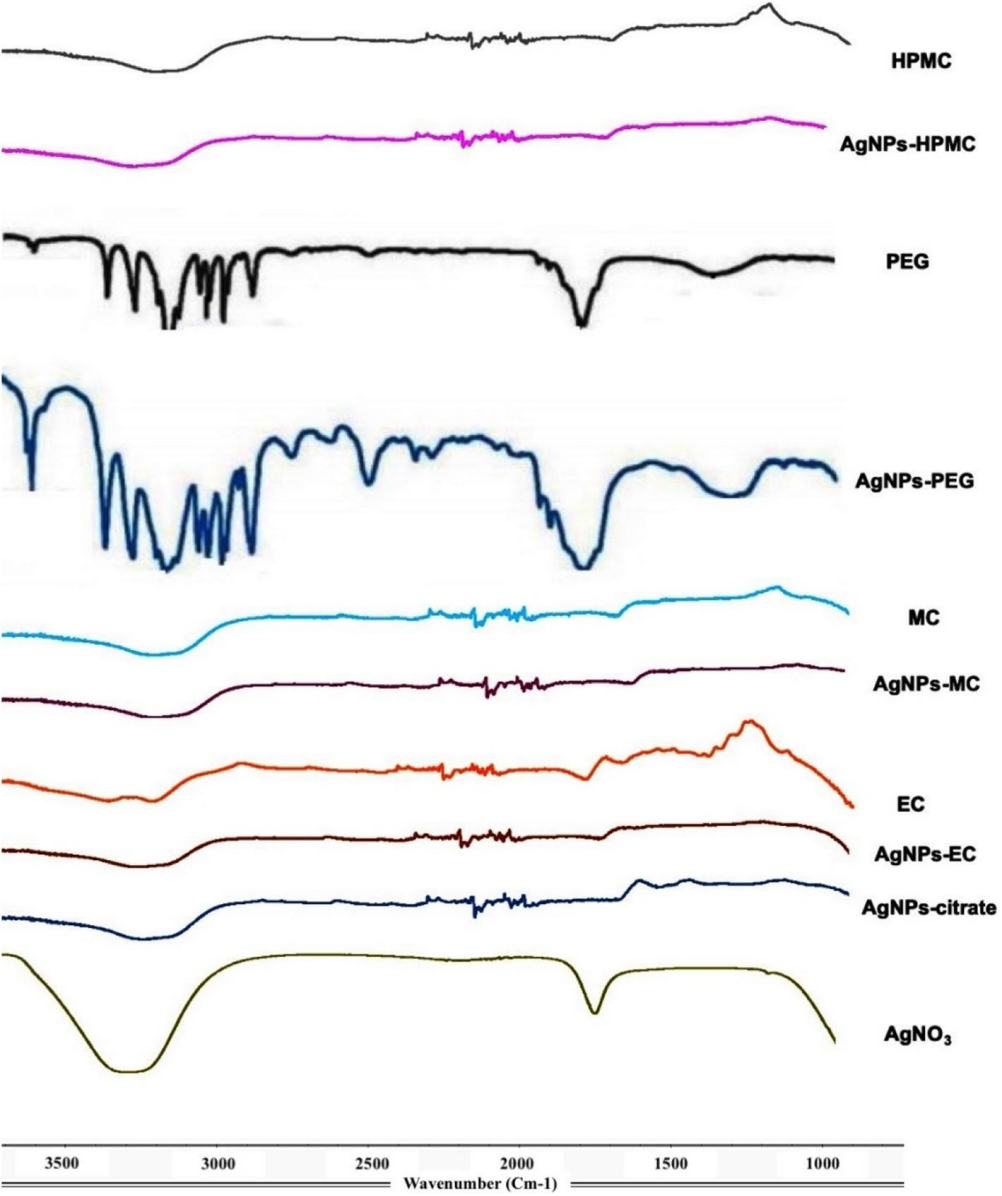
FT-IR transmission spectra of AgNPs synthesized using different polymers. From top to bottom, AgNPs reduced with methylcellulose, ethylcellulose, polyethylene glycol 6000, and hydroxypropylmethylcellulose and the respective polymers alone.

The functional groups of AgNO_3_, PEG, EC, and HPMC with AgNPs-PEG, AgNPs-EC, AgNPs-MC, and AgNPs-HPMC were verified to confirm the coating of the polymers with AgNPs (Fig. 2). FT-IR spectroscopy confirmed the formation of a coat covering the AgNPs to prevent their aggregation. The peaks in the region between 3423 and 3200 cm^−1^ were attributed to the O‒H stretching bonds of alcohol (OH group), and the aldehyde C‒H stretching of alkanes. The peak at 2923 cm^−1^ indicated the C‒H stretching of the alkane group. The peaks in the region 1610 cm^−1^ correspond to the C = C medium-weak stretching vibration of arenes, and the peaks between 660 and 770 cm^−1^ were assigned to O–H (H-bonded). The peaks obtained indicated reducing polymers on the surface of each AgNPs. The broad C–O variable weak bending vibrations of alcohols and phenols indicate that both C-O and OH groups may be responsible for the synthesis and stabilization of AgNPs. These results are in agreement with those obtained by Nunes et al.^26^. FT-IR confirmed that the characteristic groups of the reducing polymeric materials has a strong ability to bind with metal, which suggests the formation of a coating layer around AgNPs to prevent their aggregation and, hence, resulting in a higher physical stability^27^.

#### Size and zeta (ζ) potential

**Figure 3:**
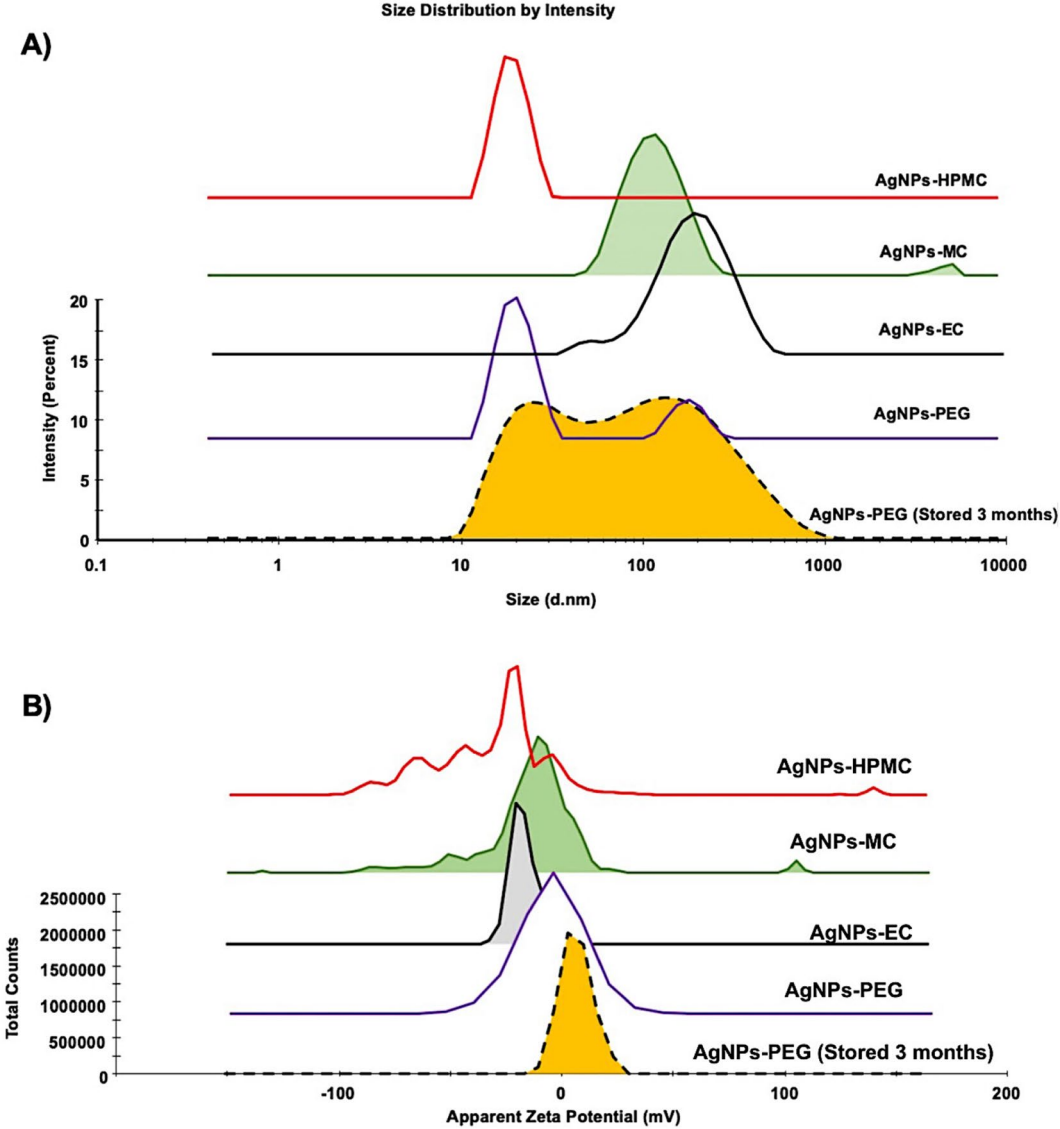
The mean diameter (nm) and ζ potential (mV) of the prepared AgNPs. The pH of the measured samples was equal to 8.0 and NPs dispersion pH and ionic strength were adjusted using 10 mM NaOH.

The geometrical size values showed uniform AgNPs with sizes 88.4 ± 0.1, 163.0 ± 0.09, 121.1 ± 0.1, and 78.55 ± 0.2 nm (Fig. 3A) and negative surface charges of ‒11.3 ± 0.5, ‒16.7 ± 0.2, ‒23.45 ± 0.6, and ‒33.5 ± 0.9 mV for AgNPs-PEG, AgNPs-EC, AgNPs-MC, and AgNPs-HPMC, respectively (Fig. 3B). The parameters of size and ζ potential indicate the stability of AgNPs. The size of all of the produced AgNPs was high due to the outer layer of the coated polymer^16^. AgNPs-MC and AgNPs-HPMC are considered more stable than AgNPs-PEG and AgNPs-EC since they showed higher ζ potential values. Barhoum et al.^28^ reported that AgNPs with ζ potentials ranging from − 10 to + 10 mV are considered neutral. However, those with ζ potential values larger than + 10 mV or less than − 10 are considered stable NPs.

#### Scanning electron microscopy

**Figure 4:**
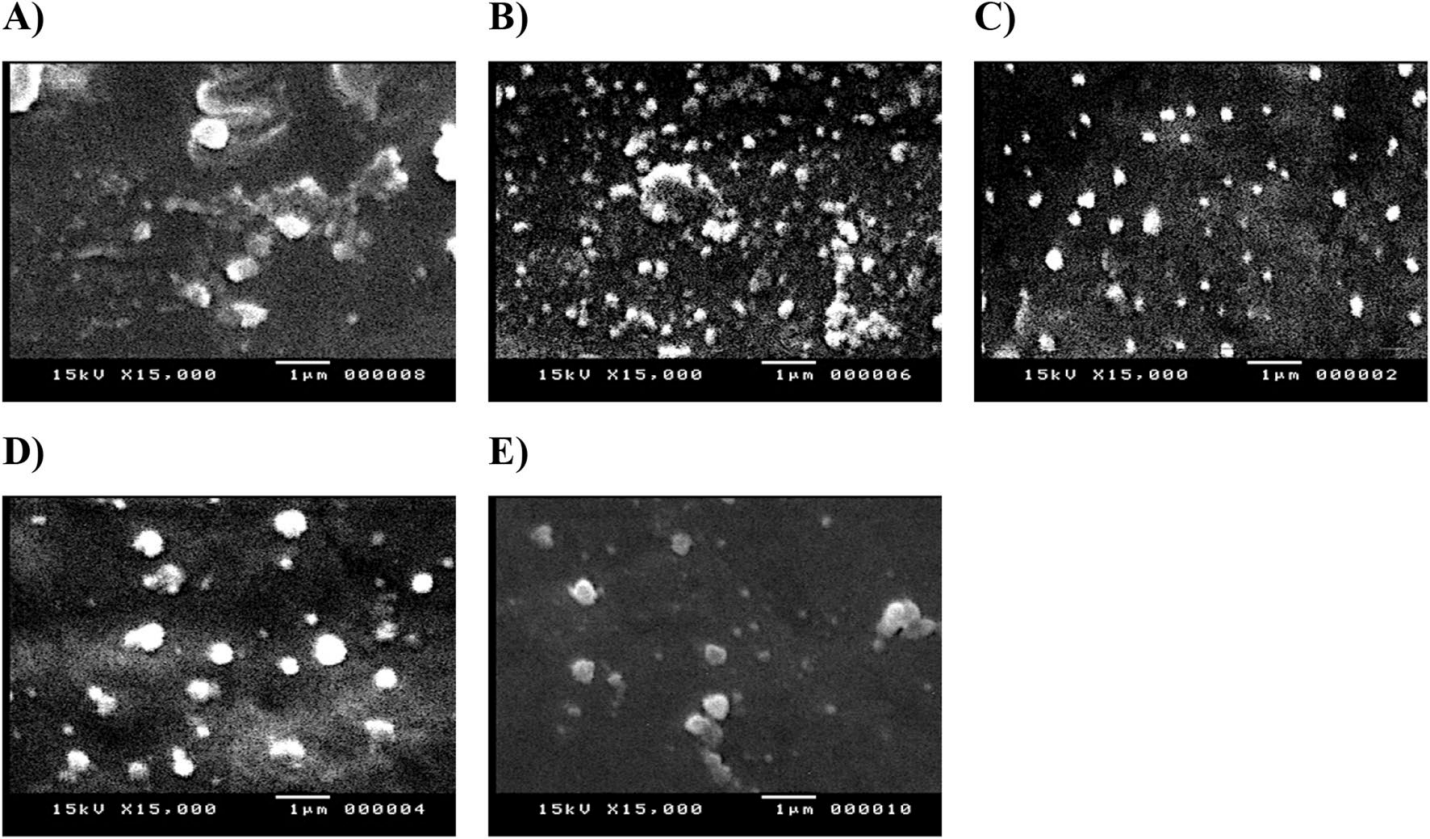
SEM images of the prepared AgNPs. (**A**) AgNPs-PEG; (**B**) AgNPs-EC; (**C**) AgNPs-MC; (**D**) AgNPs-HPMC; (**E**) AgNPs-CIT (citrate). Images are presented in magnification of 10,000 × and scale bar of 1 µm.

AgNPs formulations can appear as a very small discrete entities, either as individual or aggregated lumps of NPs with regular or irregular morphologies (Fig. 4). In the present study, AgNPs-PEG showed large, aggregated, rectangular fibrous cluster structure particles (Fig. 4; image A), which in agreement with the DLS results. AgNPs-EC, AgNPs-MC, and AgNPs-HPMC showed highly spotted nanoparticles with more uniform and non-aggregated particles (Fig. 4; images B–D). This observation also confirmed the results obtained from DLS study. AgNPs-CIT appeared as irregular, spherical particles with relatively larger particles or clusters of particles, indicating some aggregation (Fig. 4; image E). The uniform morphology of cellulosic AgNPs possibly be attributed to interactions between Ag^+^ and the C-O or OH groups located on the coated polymers. This interaction would decrease the mobility of metallic cations, prevent the growth of large particles, and stabilize the produced AgNPs^27,29^. Further morphological and size manipulations were investigated using TEM observation.

#### Transmission electron microscopy

**Figure 5:**
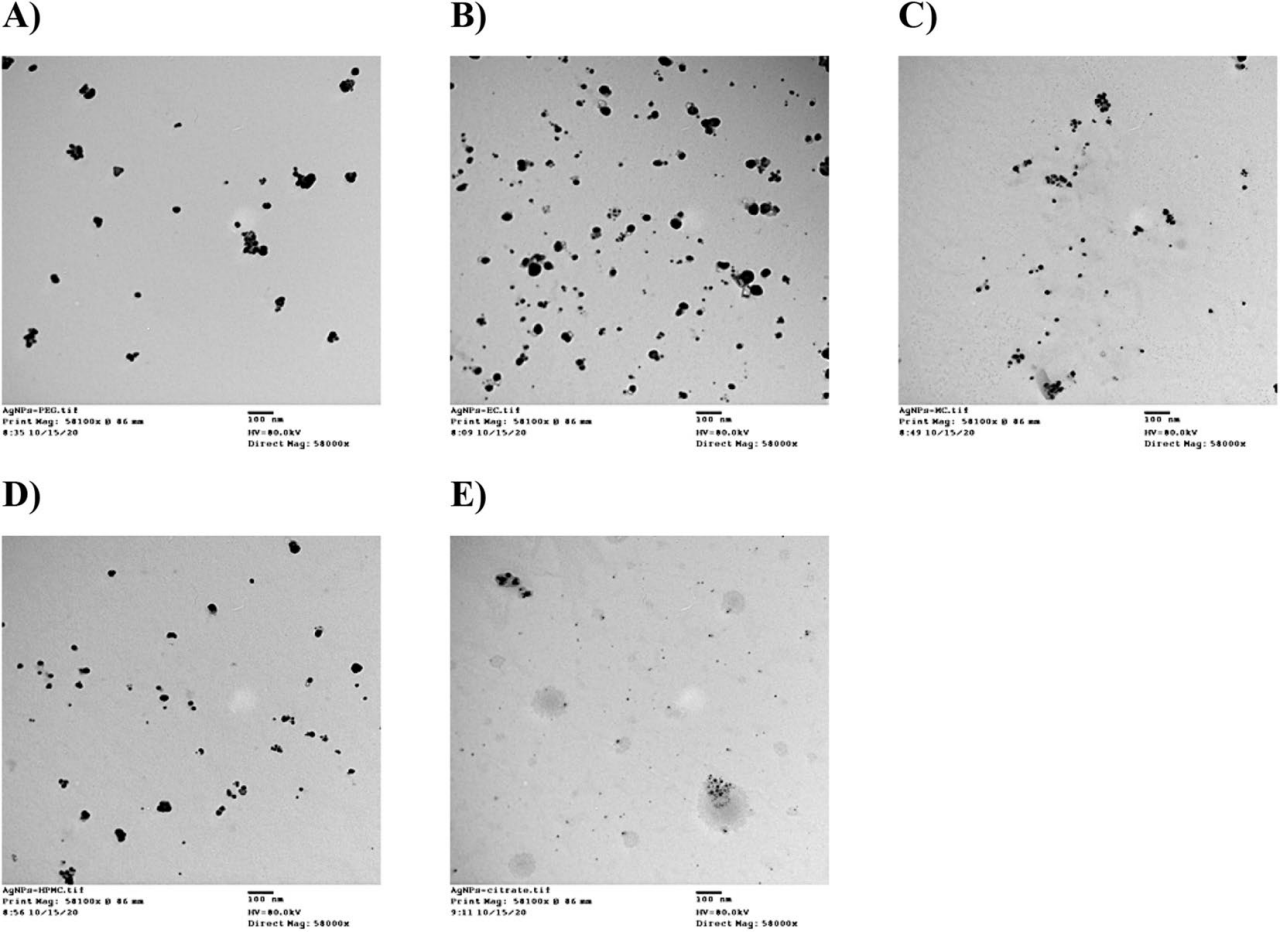
TEM images of the prepared AgNPs. (**A**) AgNPs-PEG; (**B**) AgNPs-EC; (**C**) AgNPs-MC; (**D**) AgNPs-HPMC; (**E**) AgNPs-CIT (citrate). Images are presented in magnification of 58,000 × and scale bar of 100 nm.

According to TEM, all of the AgNPs produced had spherical shapes, with an average diameter of 24.2 ± 3.6, 54.3 ± 11.6, 22.6 ± 2.6, 23.4 ± 1.3, and 11.2 ± 1.8 nm for AgNPs-PEG, AgNPs-EC, AgNPs-MC, AgNPs-HPMC, and AgNPs-citrate, respectively (Fig. 5). These results were in agreement with those obtained from DLS and SEM. However, the sizes observed via the TEM measurements were significantly lower than those obtained using the DLS technique and similar results have been observed with other previous studies^29–31^. Generally, electronic images reflect the metallic centers of the particles; TEM showed the core of the NPs as the metal core^32^. However, DLS-based measurements depend on the mean size hydrodynamic diameter. In addition, small aggregates in nanosuspension could also be measured, affecting the regular size distribution^31^.

#### In vitro silver ion release

**Figure 6:**
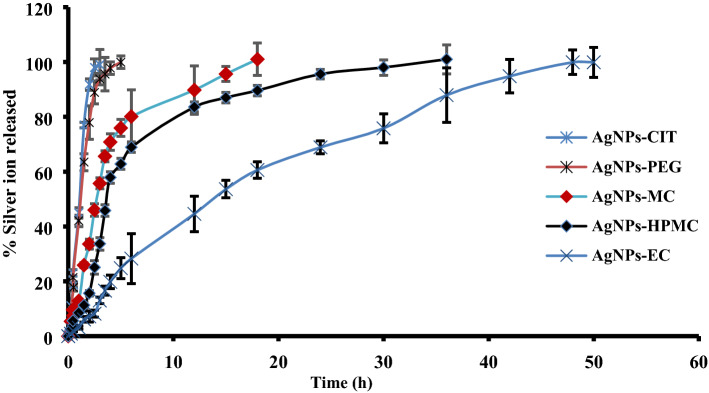
In vitro release of Ag^+^ from the prepared AgNPs-CIT, AgNPs-PEG, AgNPs-MC, AgNPs-HPMC, and AgNPs-EC in deionized water (n = 3 ± S.D.).

Ag^+^ released from different types of AgNPs was evaluated in deionized water in a hydrolytic environment. It was observed that Ag^+^ was released continually in aqueous solutions. The initial concentrations of each formulation are provided in Table 1. Figure 6 shows that the Ag^+^ content of AgNPs-CIT and AgNPs-PEG was completely released after 2.5 and 5 h, respectively. Moreover, the release of Ag^+^ from AgNPs-MC, AgNPs-HPMC, and AgNPs-EC continued for 18, 36, and 48 h, respectively. By contrast, Ag^+^ was released very quickly from AgNPs-CIT and AgNPs-PEG due to the high solubility of citrate and PEG in water. Furthermore, Ag^+^ was found to be continually released, depending on the degree of the solubility of MC, HPMC, and EC in water. Since EC is practically water insoluble, its dissolution was prolonged to 48 h^33,34^. The slower Ag^+^ release at the start of degradation can be explained by the polymer film’s shielding action, which reduces the nanoparticle‒water interaction. As a result, particle corrosion is initially regulated by the diffusion of the polymers in water, resulting in a slow cation release. The ICP-OES measurements showed that the depletion of polymer around AgNPs increased the rate of release of Ag^+^^35^.

### Physical stability

The physical stability of all the prepared AgNPs was studied for 3 months at 25 ± 0.5 °C and 4.0 ± 0.5 °C. The results showed no change in the color or morphology of the AgNPs under the two conditions investigated (25 ± 0.5 °C and 4.0 ± 0.5 °C). Moreover, a non-significant (*p* ≥ 0.05; ANOVA/Tukey) change in particle size and ζ potential was observed compared to previously prepared AgNPs. The AgNPs also showed the same wavelength, with negligible changes. However, the mean size of AgNPs-PEG increased with the appearance of a small second peak at 110 ± 11.2 nm concomitant with a lower zeta potential − 0.5 ± 0.22 (Fig. 3A,B). The decrease in the zeta potential value suggests that AgNPs-PEG would form a large aggregate. These results are in agreement with those obtained by Fernando et al.^36^, who reported that AgNPs remained stable for a long period of time after treatment with cetyl trimethyl ammonium bromide, tween-20, polyvinyl pyrrolidone, and PEG. In addition, Shi et al.^37^ found that the stability of AgNPs was significantly enhanced by coating with cellulose nanocrystals, with antibacterial activities that were enhanced four-fold, based on antibacterial studies using *Escherichia coli* and *Bacillus subtilis*. Lastly, all of the AgNPs prepared using the cellulosic polymers were found to remain stable for 3 months at both of the temperatures investigated.

### Antioxidant activity of polymeric AgNPs

**Figure 7:**
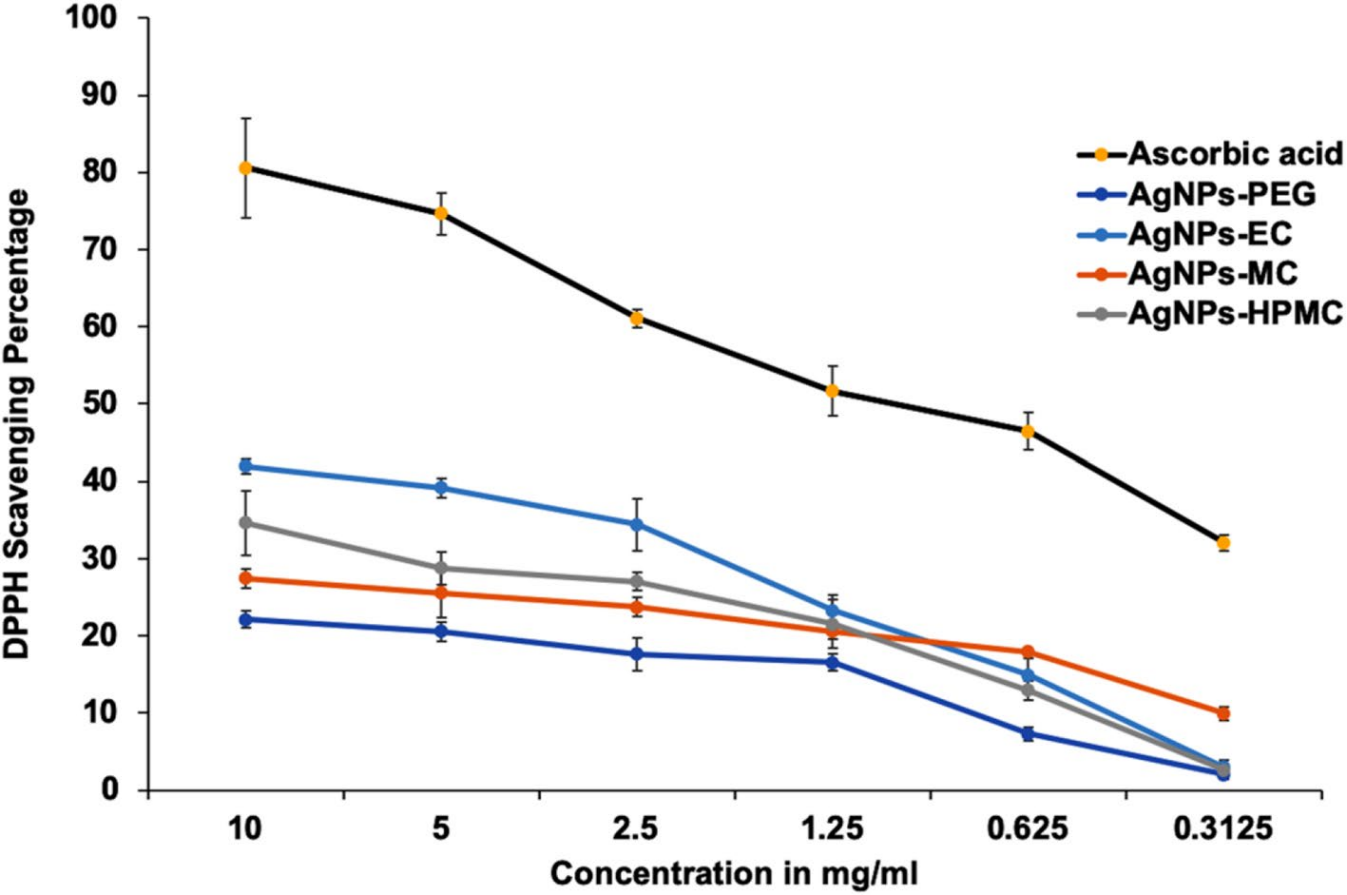
The antioxidant activity of AgNPs was presented as the DPPH scavenging percentage compared with ascorbic acid (n = 3 ± S.D.).

The antioxidant activity of AgNPs has been studied widely^38^. In addition, it was also investigated for AgNPs prepared via biological, physical, and chemical methods^39,40^. The antioxidant activity of AgNPs is very interesting when used in clinical applications, with properties such as anti-cancer activity, and the ability to provide protection against degenerative Alzheimer’s disorder^41^. The antioxidant activity of the produced AgNPs was compared with that of ascorbic acid (standard), which has a strong antioxidant activity (Fig. 7). At a lower concentration of 0.3 mg/mL, little action was observed for the investigated AgNPs on the DPPH scavenging percentage, except for AgNPs-MC, which showed a DPPH scavenging activity of 10 ± 0.6% compared to the standard ascorbic acid 32 ± 1.1%. At a concentration of 2.5 mg/mL, ascorbic acid has a DPPH scavenging activity of 61 ± 1.6%, while AgNPs prepared by HPMC and MC has a DPPH scavenging activity of 20 ± 1% and 25 ± 0.8%, respectively, and AgNPs-PEG has a DPPH scavenging activity of 18 ± 0.61%. At higher concentrations of 10 mg/mL, ascorbic acid has a DPPH scavenging activity of 80 ± 2.1% while AgNPs-EC has a DPPH scavenging activity percentage above 40 ± 1.3%^42^. AgNPs-EC showed a significantly (*p* ≤ 0.05; ANOVA/Tukey) higher antioxidant activity than AgNPs-MC and AgNPs-PEG, but had a non-significant effect compared to AgNPs-HPMC. AgNPs stabilized with EC and HPMC showed the highest antioxidant activity compared to the other polymers. In addition, they showed approximately 30 ± 1.2–40 ± 1.1% of the antioxidant activity of ascorbic acid. It was reported that the DPPH free radical was reduced by AgNPs via accepting or donating electrons, which were responsible for the color changes and formation of the hydrazine molecule^23^. Both EC and HPMC have shown antioxidant properties, as previously reported^43^. The detectable antioxidant activity of AgNPs stabilized by cellulosic polymers could be possibly attributed to the combined action of AgNPs and the cellulosic polymers.

### Antimicrobial activities

#### Screening of antibacterial activity

**Figure 8:**
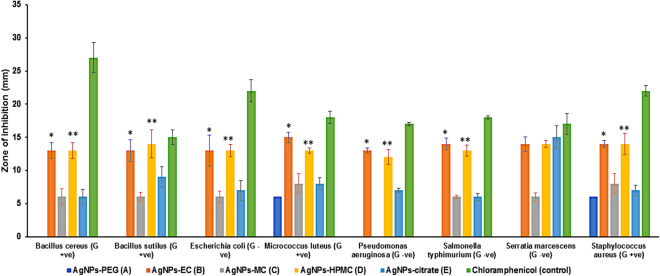
Sensitivity of the investigated bacterial species to different types of AgNPs, shown as the inhibition zone in (mm ± S.D.). AgNPs-EC, AgNPs-MC, and AgNPs-HPMC were tested at concentrations of 341 µM/mL. AgNPs-PEG and AgNPs-CIT were tested at a concentration of 1000 µM/mL. *AgNPs-EC, significant difference (*p* < 0.05; ANOVA/Tukey) versus AgNPs-MC, AgNPs-PEG, and AgNPs-citrate; **AgNPs-HPMC, significant difference (*p* < 0.05; ANOVA/Tukey) versus AgNPs-MC, AgNPs-PEG, and AgNPs-citrate.

The antibacterial activity of the investigated colloidal solutions of AgNPs was evaluated against 4 different Gram-positive and 4 different Gram-negative bacterial species. AgNPs prepared using sodium citrate were used as a control, and chloramphenicol was used as a positive antibacterial control. The antibacterial activity was presented as the diameter of the inhibition zone (mm). A higher antibacterial effect was correlated to a large clear area around the performed well. An initial screening test showed that AgNPs stabilized using EC and HPMC showed a significant (*p* ≤ 0.05; ANOVA/Tukey) antibacterial activity against the studied species of bacteria compared to AgNPs stabilized with MC and PEG, as well as those prepared without cellulosic stabilizer (AgNPs prepared using sodium citrate) (Fig. 8). In addition, AgNPs prepared using EC or HPMC did not show any significant (*p* ≥ 0.05; ANOVA/Tukey) antibacterial difference against the tested Gram-positive and Gram-negative bacteria.

#### The sensitivity of different bacterial species to different concentrations of AgNPs

Different concentrations of AgNPs were tested for their antibacterial activity in eight species of bacteria (Table 2A-C, Fig. 8). AgNPs prepared using PEG and sodium citrate were used at high concentrations (1000 and 500 µM/mL). However, other AgNPs were tested at lower concentrations ranging from 341 to 5.33 µM/mL. The variations in the tested concentrations depended on the preparation method of each type of AgNPs. AgNPs stabilized with PEG (Table 2A) showed only lower antibacterial activity towards *Micrococcus luteus* and *Staphylococcus aureus* at high AgNPs concentrations (1000 µM/mL). However, they did not show any antibacterial activity towards the other Gram-positive bacteria and the tested Gram-negative bacteria at the investigated concentrations. AgNPs prepared using sodium citrate without any cellulosic stabilizer (Table 2C) showed variable activity against both Gram-positive and Gram-negative bacteria at a higher concentration of 1000 µM/mL without any effect at a lower concentration of 500 µM/mL. A higher antibacterial effect was observed with *Bacillus subtilis* and *Serratia marcescens* as a Gram-positive and Gram-negative bacterial species, respectively. Moreover, AgNPs stabilized with MC (Table 2B) showed some antibacterial activity at a high concentration of 341 µM/mL, which was more pronounced with Gram-positive bacterial species, such as *Staphylococcus aureus* and *Micrococcus luteus*.

**Table 2:** Sensitivity of bacterial species to different concentrations of AgNPs, shown as inhibition zone in (mm ± S.D.) Table 2A shows AgNPs-PEG and AgNPs-EC; Table 2B shows AgNPs-MC; Table 2C shows AgNPs-HPMC and AgNPs-citrate. Concentrations are presented in µM/mL (‒), not determined as the previous concentration showed zero activity.

(A) Sample type and conc	AgNPs-PEG		AgNPs-EC						
Bacterial strain	1000	500	341	170.5	85.25	42.62	21.31	10.65	5.33
*Bacillus cereus*	0	–	13 ± 1.2	12 ± 0.7	12 ± 0.7	10 ± 1.7	8 ± 0.3	6 ± 0.6	0
*Bacillus subtilis*	0	–	13 ± 1.6	13 ± 0.4	10 ± 1.4	8 ± 0.4	8 ± 1.2	0	–
*Escherichia coli*	0	–	13 ± 2.3	11 ± 1.2	10 ± 0.4	10 ± 1.5	8 ± 1.5	0	–
*Micrococcus luteus*	6 ± 0.7	0	15 ± 0.8	12 ± 0.9	11 ± 0.9	9 ± 0.8	8 ± 0.8	0	–
*Pseudomonas aeruginosa*	0	–	13 ± 0.4	13 ± 0.4	13 ± 1.5	10 ± 0.9	9 ± 0.9	0	–
*Salmonella typhimurium*	0	–	14 ± 0.9	14 ± 0.3	14 ± 1.6	10 ± 2.2	10 ± 1.1	0	–
*Serratia marcescens*	0	–	14 ± 1.1	14 ± 0.6	14 ± 0.5	10 ± 1.3	10 ± 0.3	0	–
*Staphylococcus aureus*	6 ± 0.9	0	14 ± 0.5	12 ± 1.3	10 ± 0.2	8 ± 0.5	6 ± 0.7	0	–

(B)	AgNPs-MC								
	341	170.5	85.25	42.62	21.31				
*Bacillus cereus*	6 ± 1.2	6 ± .0.2	0	–	–				
*Bacillus subtilis*	6 ± 0.7	0	–	–	–				
*Escherichia coli*	6 ± 0.9	6 ± 0.7	0	–	–				
*Micrococcus luteus*	8 ± 1.5	0	–	–	–				
*Pseudomonas aeruginosa*	0	–	–	–	–				
*Salmonella typhimurium*	6 ± 0.3	6 ± 1.1	6 ± 0.9	6 ± 0.2	0				
*Serratia marcescens*	6 ± 0.6	6 ± 0.8	6 ± 0.6	6 ± 0.4	0				
*Staphylococcus aureus*	8 ± 1.4	0	0	–	− 0				

(C)	AgNPs-HPMC							AgNPs-CIT	
	341	170.5	85.25	42.62	21.31	10.65	5.33	1000	500
*Bacillus cereus*	13 ± 1.2	12 ± 0.7	10 ± 0.4	0	–	–	–	6 ± 1.1	0
*Bacillus subtilis*	14 ± 2.1	11 ± 0.4	10 ± 0.2	0	–	–	–	9 ± 1.6	0
*Escherichia coli*	13 ± 0.9	10 ± 1.2	10 ± 0.9	6 ± 1.1	0	–	–	7 ± 1.5	0
*Micrococcus luteus*	13 ± 0.4	12 ± 1.4	11 ± 1.1	0	–	–	–	8 ± 0.9	0
*Pseudomonas aeruginosa*	12 ± 1.1	12 ± 0.4	12 ± 0.7	0	–	–	–	7 ± 0.3	0
*Salmonella typhimurium*	13 ± 0.8	13 ± 0.2	13 ± 1.2	6 ± 0.3	6 ± 0.4	6 ± 0.9	0	6 ± 0.5	0
*Serratia marcescens*	14 ± 0.5	14 ± 0.5	14 ± 1.1	6 ± 0.2	6 ± 0.6	0	–	15 ± 1.7	0
*Staphylococcus aureus*	14 ± 1.6	10 ± 0.8	8 ± 0.9	0	–	–	–	7 ± 0.8	0

Regarding AgNPs stabilized with EC and HPMC (Table 2A&C), they showed antibacterial activity in a concentration gradient profile for all the investigated bacterial species. HMPC-stabilized AgNPs showed an antibacterial activity up to a concentration of 85.25 µM/ml. However, AgNPs stabilized with EC showed an antibacterial activity with a smaller concentration, of 21.31 µM/mL, respectively (Table 2A). It is also worth noting that AgNPs stabilized with either EC or HPMC showed a more pronounced antibacterial effect on Gram-negative bacterial species, such as *Pseudomonas aeruginosa*, *Salmonella typhimurium*, and *Serratia marcescens*, compared with the other tested Gram-positive bacterial species at a lower concentration, of 21.31 and 85.25 µM/ml, respectively. In addition, AgNPs stabilized with EC and HPMC had almost similar antibacterial effect to chloramphenicol in terms of their effect on *Bacillus subtilis* and *Serratia marcescens*.

#### Minimum inhibitory concentration

The MIC for the produced AgNPs, expressed as µM/mL, with the antibacterial effect as an inhibition zone (mm ± SD) is presented in Table 3, S1 (Images of the agar plates). Generally, lower MICs were recorded for AgNPs stabilized with EC and HPMC, followed by MC and AgNPs prepared using sodium citrate and AgNPs stabilized with PEG. AgNPs prepared using EC had an MIC of 21.31 µM/mL for all the tested bacterial species with an MIC of 10.65 µM/mL for *Bacillus aureus*. Whereas, AgNPs stabilized with HPMC have a MIC of 85.25 µM/mL for the tested Gram-positive bacterial species, and 85.25, 42.62, 21.31, and 10.65 µM/mL for *Pseudomonas aeruginosa*, *Escherichia coli*, *Serratia marcescens*, and *Salmonella typhimurium*, respectively. AgNPs stabilized using MC have a lower MIC value (42.62 µM/mL) towards *Serratia marcescens* and *Salmonella typhimurium* as Gram-negative bacteria, an intermediate MIC value of 170.5 µM/mL for *Bacillus cereus* and *Escherichia coli*, and a higher MIC of 341 µM/mL for *Bacillus subtilis*, *Micrococcus luteus*, and *Staphylococcus aureus* as a Gram-positive bacterium.

**Table 3:** Antibacterial activity expressed as inhibition zone (mm ± S.D.) and MICs (in μM/mL, given in brackets) of AgNPs reduced with cellulosic polymers compared with PEG and AgNPs prepared using tri-sodium citrate. A: AgNPs-PEG; B: AgNPs-EC; C: AgNPs-MC; D: AgNPs-HPMC; E: AgNPs-citrate. (‒), not determined as the previous concentration showed zero activity.

Sample type Bacterial strains	A	B	C	D	E
*Bacillus cereus*	–	6 ± 0.1	6 ± 0.3	10 ± 0.5	6 ± 0.8
		(10.65)	(170. 5)	(85. 25)	(1000)
*Bacillus subtilis*	–	8 ± 0.3	6 ± 0.1	10 ± 0.6	9 ± 1.0
		(21.31)	(341)	(85. 25)	(1000)
*Escherichia coli*	–	8 ± 0.2	6 ± 0.2	6 ± 0.4	7 ± 0.5
		(21.31)	(170. 5)	(42.62)	(1000)
*Micrococcus luteus*	6 ± 0.2	8 ± 0.2	8 ± 0.2	11 ± 0.7	8 ± 0.4
	(1000)	(21.31)	(341)	(85. 25)	(1000)
*Pseudomonas aeruginosa*		9 ± 0.1	–	12 ± 0.9	7 ± 0.3
	–	(21.31)		(85. 25)	(1000)
*Salmonella typhimurium*		10 ± 0.3	6 ± 0.3	6 ± 0.7	6 ± 0.5
	–	(21.31)	(42.62)	(10.65)	(1000)
*Serratia marcescens*		10 ± 0.2	6 ± 0.4	6 ± 0.5	15 ± 0.9
	–	(21.31)	(42.62)	(21.31)	(1000)
*Staphylococcus aureus*	6 ± 0.1	6 ± 0.4	8 ± 0.6	8 ± 0.9	7 ± 0.8
	(1000)	(21.31)	(341)	(85. 25)	(1000)

Finally, it could be concluded that different cellulosic polymers could significantly affect the bacterial activity of the formulated AgNPs. Cellulosic stabilizers showed a significant (*p* ≤ 0.05; ANOVA/Tukey) antibacterial effect compared to PEG and AgNPs prepared using sodium citrate^27^. In addition, AgNPs stabilized via EC have lower MIC values compared to AgNPs stabilized with HPMC against the investigated Gram-positive and Gram-negative bacteria. It is worth mentioning that AgNPs formulated using EC were highly active against *Bacillus cereus* as Gram-positive bacteria and *Salmonella typhimurium*, *Serratia marcescens*, and *Pseudomonas aeruginosa* as Gram-negative bacteria.

Generally, AgNPs have proven antibacterial action, the expected mechanism of which was reported by Rajeshkumar et al.^44^ through the release of silver ions, which breaks the cell wall, disrupting protein synthesis, resulting in microbial death. AgNPs rather attack the bacterial respiratory chain, while cell division finally leads to cell death. Other studies have revealed that AgNPs can produce reactive oxygen species and superoxide anions. Amro et al.^45^ reported that the formation of asymmetrical pits in the external membrane can cause metal exhaustion and modify membrane permeability induced by a gradual release of membrane proteins and lipopolysaccharides. In this study, it was clear that different activities against bacterial species could be attributed to the type and method of AgNPs preparation as well as the coating material^46^. AgNPs prepared through the conventional reduction method using sodium citrate showed antibacterial activity at high concentrations only which was expected due to the absence of a stabilizer and coating agent; hence, particles may show some aggregation, which finally affects the size. PEG-stabilized AgNPs showed the lowest activity against the investigated bacterial species even though they were examined at higher concentration^47^ which could possibly be attributed to high linked Ag^+^^47^. The higher antibacterial activity of EC and HPMC stabilized AgNPs could be attributed to disrupting the cellular metabolic processes of bacteria^48^. A similar study revealed a higher antibacterial effect of AgNPs stabilized with HMPC than AgNPs prepared using MC and HEC^46^. The broad activity towards the gram-negative strains could be attributed to the difference between the structure of the membrane of gram-negative and positive bacteria represented in the thickness of peptidoglycan layer^49^. Investigation of the effect of cellulosic AgNPs on multidrug-resistant clinical strains showed superior action against *Escherichia coli* and *Staphylococcus aureus*. The MIC values for *Escherichia coli* were 42.62 ± 10.0 µM/mL for the three types of cellulosic AgNPs. Whereas, 21.3 ± 5.0, 42.6 ± 10.0 and 21.3 ± 5.0 µM/mL for AgNPs-EC, AgNPs-MC and AgNPs-HPMC, respectively regarding *Staphylococcus aureus*. It was also found that MIC values were significantly (*p* ≤ 0.05; ANOVA/Tukey) lower regrading AgNPs-MC for both clinically isolated bacterial strains compared to standard strains. However, it was significantly lower (*p* ≤ 0.05; ANOVA/Tukey) in cases of *Staphylococcus aureus* using AgNPs-HPMC. This observation could be due to the higher permeability of the cell wall of *Staphylococcus aureus* clinical isolates than standard strains, which allow more penetration of AgNPs through the cell wall^50^. Thus, finally reflected the higher efficiency of the formulated AgNPs against these types of isolates, which are able to form a highly resistant biofilm^51^ which deserves further investigation for other resistant bacterial strains.

The obtained AgNPs-polymers are to be used safely, as they are prepared from natural safe polymers. These results agree with those of Zahran et al. who reported that the preparation of AgNPs using sodium alginate is non-toxic and ecofriendly for stable/well obtained AgNPs^52^. Moreover, natural polymers reduced with AgNO_3_ to form AgNPs preparations have been considered to obtain safer NPs via a green chemistry approach^13,14^.

### Silver NPs quantification in bacterial cells

**Figure 9:**
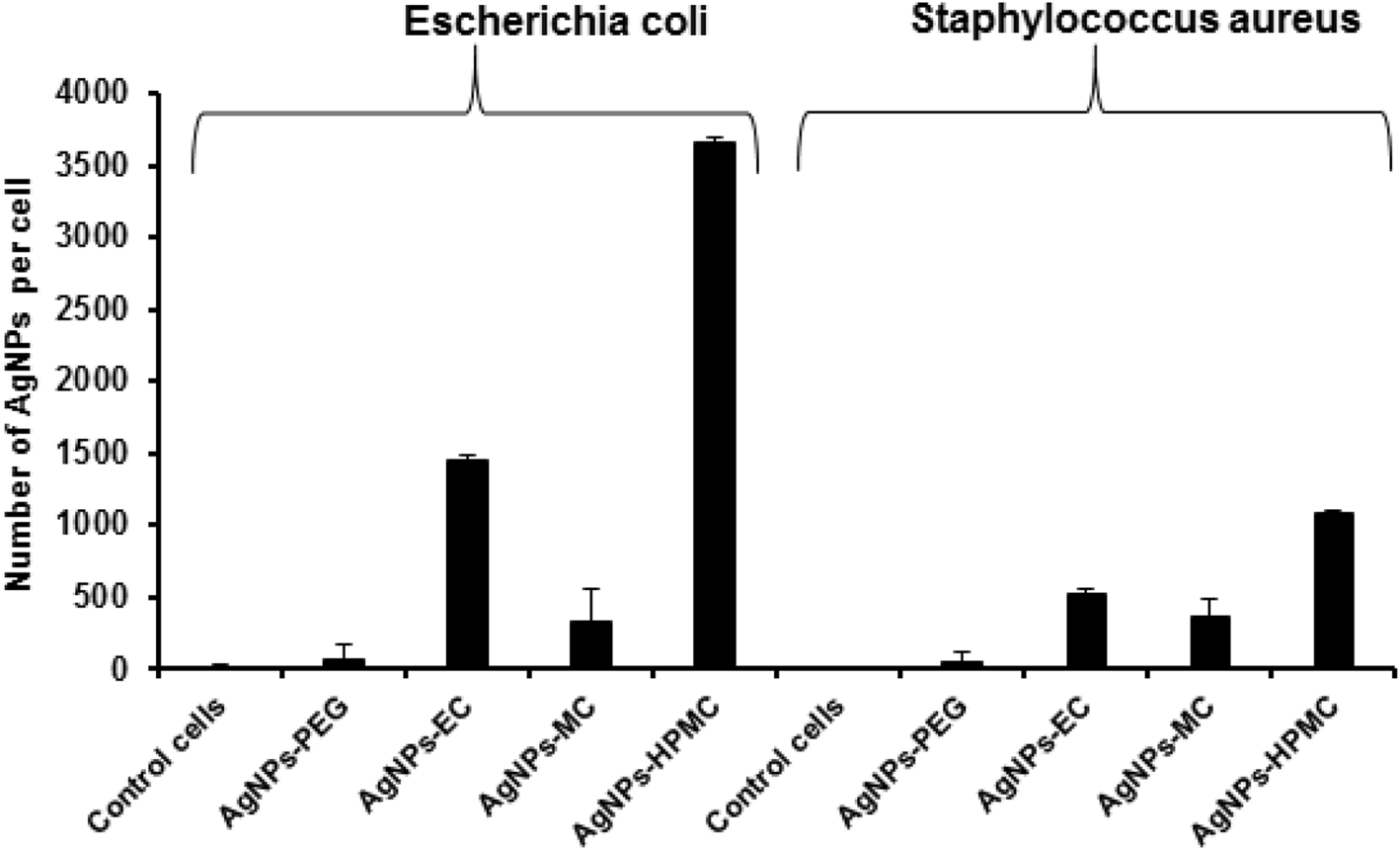
ICP-OES for AgNPs represents the amount of AgNPs internalized into *Escherichia coli* and *Staphylococcus aureus*. AgNPs-PEG (500 µM/mL), AgNPs-EC (42.62 µM/mL), AgNPs-MC, and AgNPs-HPMC (85.25 µM/mL). Control cells were used as negative controls.

A quantitative study as a support to corroborate the antibacterial studies of different classes of polymeric AgNPs was conducted using ICP-OES, which can identify how many AgNPs have been imbedded/incorporated inside the bacterial interior, causing breakage in the electron transport chain and inhibition of the intrinsic protein synthesis machinery and, hence, DNA damage of the chosen prokaryotic bacterial species. Cellulosic stabilized AgNPs were efficiently internalized into the studied bacterial strains with a significant NPs number (*p* ≤ 0.05; ANOVA/Tukey) compared with AgNPs-PEG and control cells (Fig. 9). The number of AgNPs incorporated inside the *Escherichia coli* was found to be 1447 ± 115.6, 335 ± 45.5, and 3660 ± 27.5 NPs/cell for AgNPs-EC, AgNPs-MC, and AgNPs-HPMC, respectively, compared to 59.8 ± 15.3 NPs/cell for AgNPs-PEG. In addition, the number of AgNPs incorporated inside the *Escherichia coli* was significantly higher (*p* ≤ 0.05; ANOVA/Tukey) than those inside *Staphylococcus aureus*, 520.9 ± 69.4, 363.3 ± 20.5, and 1080 ± 120.6 NPs/cell for AgNPs-EC, AgNPs-MC, and AgNPs-HPMC, respectively. This result is in accordance with the antimicrobial activity study shown earlier regarding the higher activity of the formulated cellulosic AgNPs towards the gram-negative bacteria than the gram-positive one. It is also worth mentioning that, despite the low concentration of AgNPs-EC, 42.62 µM/ml, it showed high NPs internalized per bacterial cell with both types of bacterial strains. AgNPs-PEG showed negligible internalization in both studied bacterial strains, which is also in agreement with the results obtained from the MIC study. Despite of the smaller NPs size of AgNPs-PEG but they showed a negligible internalization due to their aggregation and possibly high M.wt of PEG (6000 Da). However, HPMC and EC have M.wt of 1261.4 and 454.5 g/mol, respectively, and their AgNPs formulations can resist aggregation; hence, higher NPs internalization. The highest AgNPs embedded inside the bacterial strains for AgNPs-HPMC could possibly be attributed to the higher penetration of HPMC into the bacterial cell membrane^53^, high NPs yield, and continuous release of Ag^+^ for a long period of time^54^.

### Morphological observation of bacterial cells after AgNPs internalization

**Figure 10:**
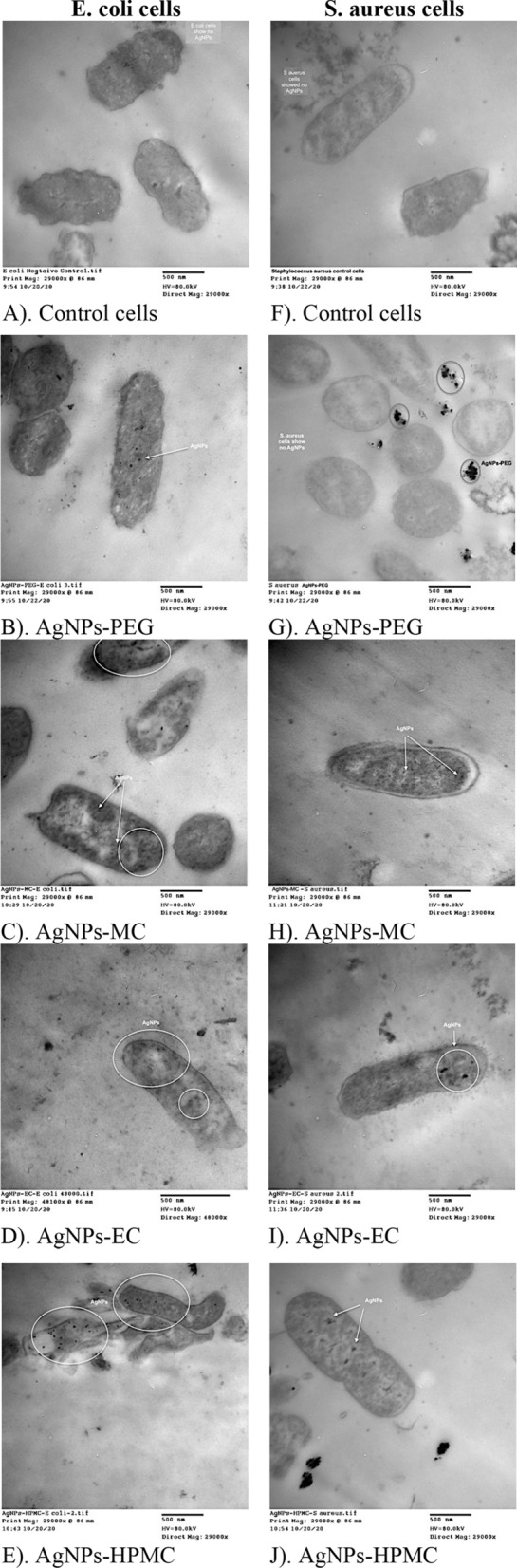
TEM micrographs of AgNPs internalized into *Escherichia coli* and *Staphylococcus aureus.* (**A**) *Escherichia coli* control cells; (**B**) AgNPs-PEG 500 µM/mL/*Escherichia coli*; (**C**) AgNPs-MC 85.25 µM/mL/*Escherichia coli*; (**D**) AgNPs-EC 42.62 µM/mL/*Escherichia coli*; (**E**) AgNPs-HPMC 85.25 µM/mL/*Escherichia coli*; (**F**) *Staphylococcus aureus* control cells; (**G**) AgNPs-PEG 500 µM/mL/*Staphylococcus aureus*; (**H**) AgNPs-MC 85.25 µM/mL/*Staphylococcus aureus*; (**I**) AgNPs-EC 42.62 µM/mL/*Staphylococcus aureus*; (**J**) AgNPs-HPMC 85.25 µM/mL/*Staphylococcus aureus*. The magnification bar represents 500 nm with a magnification power of 29,000 × and 45,000 × .

TEM micrographs showed the internalization of AgNPs after attacking the bacterial cell membrane and crossing the cell wall of the two investigated bacterial species, *Escherichia coli* and *Staphylococcus aureus* (Fig. 10). It was observed from the images that higher amounts of AgNPs stabilized with cellulosic polymers were accumulated inside the cell interior of *Escherichia coli* (Fig. 10; images C–E) and *Staphylococcus aureus* (Fig. 10; images H–J) compared with AgNPs-PEG (Fig. 10; images B and G). AgNPs-PEG showed a small number of NPs internalized inside *Escherichia coli* cells and aggregated NPs outside the cells of *Staphylococcus aureus*, which hindered their internalization (Fig. 10; images B and G, respectively). It was also interesting to observe higher amounts of AgNPs-cellulosic polymers inside *Escherichia coli* (Fig. 10; images C, D and E) compared with those found inside *Staphylococcus aureus* (Fig. 10; images H, I, and J). This finding coincides with the other investigated results, especially those obtained from AgNPs quantification via ICP-OES analysis. From a mechanistic point of view, AgNPs could interact with bacterial cell wall components such as proteins and unsaturated fatty acids, leading to inactivation of enzymes and proteins associated with the cell wall of bacteria^55,56^. Thus, followed by enhancing bacterial membrane permeability and fluidity and overall disruption of cell wall and altering membrane integrity^57^ as shown in (Fig. 10; images D and E) for AgNPs-EC and AgNPs-HPMC, respectively*, interacted with Escherichia coli*. After the internalization of AgNPs, they could interact with bacterial DNA causing disturbance and accumulation into the cytoplasm of damaged bacteria, leading to leakage of cellular components and bacterial death^58,59^.

## Materials and methods

### Materials

Silver nitrate, tri-sodium citrate, sodium borohydride, methylcellulose (MC), hydroxypropyl methylcellulose (HPMC), ethylcellulose (EC), polyethylene glycol 6000 (PEG), sodium chloride, potassium chloride, disodium hydrogen phosphate, potassium di-hydrogen phosphate, sodium hydroxide, and hydrochloric acid were purchased from VBBN Company, Hong Kong, China. *Bacillus cereus* (G + ve; AUMC No. B-52), *Bacillus subtilis* (G + ve; AUMC No. B-63), *Micrococcus luteus* (G + ve; AUMC No. B-112), *Staphylococcus aureus* (G + ve; AUMC No. B-54), *Escherichia coli* (G-ve; AUMC No. B-53), *Pseudomonas aeruginosa* (G-ve; AUMC No. B-73), *Salmonella typhimurium* (G-ve; AUMC No. B-62)*, and Serratia marcescens* (G-ve; AUMC No. B-55) were obtained and tested at the Assiut University Mycological Centre, AUMC. Clinical strains of *Escherichia coli* and *Staphylococcus aureus* were obtained from Assiut University Hospital. All other chemicals were of analytical grade, and each glassware was washed using distilled water and dried in an oven at 40 ± 1.0 °C.

### Methods

AgNPs were prepared according to the method published by Kolarova et al., 2017^60^ with some modifications. Briefly, a 1% w/v aqueous stock solution of each of the following polymers: HPMC, MC, and EC were prepared first. Then, AgNO_3_ was dissolved in 50 mL distilled water to prepare a (1 mM) solution of AgNO_3_ stock solution. 6.8 mL of AgNO_3_, which is ≅ 17% of the stock solution, was completed with distilled water to 100 mL. One mL of 10 mM NaOH was added to the prepared stock solution of AgNO_3_ solution to adjust the pH ≅ 8 and the ionic strength of solution^54^. Further, the solution was heated to ≅ 100 °C. The temperature was controlled using a mercury thermometer. Then, 2 mL of each 1% polymeric stock solution was added to the boiling AgNO_3_ stock solution. The solution was left for further 30 min under vigorous stirring. The color changed from colorless to yellowish and dark brown in color according to each polymer. The heating process was stopped and the solution was allowed to cool slowly to room temperature ≅ 30–37ºC. Each prepared sample was purified by centrifugation at 1000 rpm for 5 min. The filtrate was collected as a purified AgNPs, while the precipitate was removed, as the large particles were precipitated in the form of aggregate^54^. The purified AgNPs of each reducing agent were stored in an amber glass container at 4.0 °C for further analysis. AgNPs stabilized using PEG were prepared as previously reported, with minor changes^61^. 2 ml of AgNO_3_ stock solution was added to 48 ml of 1% PEG 6000 and stirred for 15 min in dark conditions. Then, an aqueous solution of 2 mM sodium borohydride was added dropwise until the solution color turned yellow. Afterward, the NPs solution was centrifuged at 12.000 rpm for 15 min and finally stored like the other AgNPs solutions. AgNPs-citrate was prepared as in the last procedure for AgNPs-PEG, except that 2 mL of AgNO_3_ was added to 48 mL of 1% tri-sodium citrate solution.

### Characterization of AgNPs

#### UV–Vis spectroscopy

The reduction of silver nitrate and formation of AgNPs stabilized with different stabilizers was confirmed by scanning the obtained solutions using a UV–VIS spectrophotometer (Varian, model: cary50conc, Australia) within a wavelength range of 300–600 nm. The UV–VIS absorption was analyzed after centrifugation of each particle and the redispersion of AgNPs in distilled water^62^.

#### Percentage yield of synthetized AgNPs

The amount of Ag^+^ converted to Ag^0^, which represents the % yield of the produced AgNPs according to a previously reported method with some modifications was determined^22^. Inductively Coupled Plasma Optical Emission Spectrometry (ICP-OES, iCAP 6000, Thermo Scientific, USA) was used to calculate the amount of Ag^0^ in AgNPs. 5% HNO_3_ dissolved in distilled water to facilitate the detection of free Ag^+^ at a wavelength of 324 nm. The reaction flask samples were diluted 20 × with the 5.0% HNO_3_ solution and analyzed for the total amount of Ag (AgNPs plus unreacted Ag^+^). Then, NaCl was added at the same concentration as AgNO_3_ to each sample test to precipitate Ag^+^ as AgCl, while the formed Ag^+^ of AgNPs was not precipitated. After 12 h, the AgCl was centrifuged at 2000 rpm for 13 min and the supernatant was evaluated by ICP-OES. The quantity of Ag^+^ was calculated in the supernatant, and then the total number of Ag^+^ calculated in the bio-reduced AgNPs. Each solution was scanned five times, then the % yield of the produced AgNPs was calculated by dividing the obtained concentration by the initial concentration of AgNO_3_ using the following equation:

1 $${\text{\% }}\;{\text{AgNPs}}\;{\text{ yield}} = \left[ {\frac{Concentration\; of\; each\; sample \;by \;ICP - OES}{{Initial\; Concentration \;of\; AgNO3}}} \right] \times 100$$

#### Fourier-transform infrared spectroscopy (FT-IR)

The compatibility between all components of AgNPs stabilized with the investigated polymers as well as any further interactions were investigated using an FT-IR spectrometer (Varian Company model:640-IR, Australia). Samples were produced and spectra were collected from 4000 to 400 cm^−163^.

#### Size and ζ potential

The geometrical particle size and the ζ potentials of all formulated AgNPs were determined using a Malvern Zetasizer Nano ZS Malvern Instruments GmbH (Herrenberg, Germany)^63^. In brief, an aqueous solution of each sample was adjusted to ≈25 °C and then exposed to a laser beam of ≈633 nm at a scattering angle of ≈90^°^. The obtained results were calculated as the average of the three measurements, whereas each measurement was run ≈20 times (with a ≈10 s duration)^64^.

#### Scanning electron microscopy

10 µl of each AgNPs solution was placed on the surface of the double-sided copper conductive tape and allowed to dry. The AgNPs were then sputter-coated (Sputter coater, JOEL JFC-1300) using a thin layer of platinum in a vacuum chamber for 55 s at 25 mÅ using a coating unit to make it electrically conductive before imaging in an SEM instrument. The shape of the prepared AgNPs was investigated using a scanning electron microscope (SEM, JEOL JSM-550, Japan)^31^.

#### Transmission electron microscopy

The size of the prepared NPs as well as their morphology were investigated using a transmission electron microscope (JEM-1230, Joel, Japan). Briefly, a sample of each chemically synthesized AgNPs was mounted on a carbon-coated 300 mesh copper grid by simply dropping a very small amount of the sample on the grid, followed by drying overnight. Further, samples were examined using 10–100 K magnification microscope power and an accelerating voltage of 100 kV^31,65^.

#### In vitro silver ion release

The in vitro release of silver ions from the formulated polymeric stabilized AgNPs compared with AgNPs-CIT was performed as previously described^35^. Thin films were prepared using each investigated AgNPs approximately about 3.0 cm^2^ and incubated with 50 mL deionized water for 48 h. Samples were taken at different time points for analysis of silver cation using ICP-OES after careful calibration using standard silver solution. The experiment was performed in triplicate at room temperature using an orbital shaker (WiseShak, SHO-2D, Korea) rotating at 100 rpm.

### Physical stability

The physical stability of the prepared AgNPs was tested by storing the prepared AgNPs for 3 months at 25.0 ± 0.5 °C and 4.0 ± 0.5 °C^66^. The physical characteristics such as color, morphology, particle size, and zeta potential were estimated before and after storage to check the stability of the prepared AgNPs.

### Antioxidant activity of AgNPs

The antioxidant activity of different formulations was determined in vitro using 2,2-diphenyl-1-picrylhydrazyl (DPPH)^67^. Ascorbic acid was used as a positive standard antioxidant. Briefly, 100 µL of the AgNPs prepared from the investigated reducing agents in a concentration ranging from 0.3–10 mg/mL^68^ (Ascorbic acid was used as control) were added to 1900 µL of DPPH—methanolic solution in a concentration of 300 µM. The mixtures were then strongly vortexed and set aside in a dark place for 30 min. Then, mixtures were measured at λ_max_ of 617 nm using a UV–VIS spectrophotometer, and the percentage of the DPPH free radical scavenging activity was calculated using the following equation:

2 $$\begin{aligned} \left( {x + a} \right)^{n} & = \mathop \sum \limits_{k = 0}^{n} \left( {\begin{array}{*{20}c} n \\ k \\ \end{array} } \right)x^{k} a^{n - k} \\ {\text{\% Scavenging }}\;{\text{activity}} & = 1 - \left[ {\frac{Sa}{{Ba}}} \right]x 100 \\ \end{aligned}$$

where, Sa; refers to the sample absorbance (AgNPs or ascorbic acid) and Ba is the blank absorbance.

### Antimicrobial activities

The antimicrobial activity of the prepared AgNPs was tested against 8 different bacterial species. Microbial strains were kindly provided by the Assiut University Moubasher Mycological Centre (AUMMC). The following bacterial species were selected in this study: *Bacillus cereus, Bacillus subtilis, Escherichia coli, Micrococcus luteus, Pseudomonas aeruginosa, Salmonella typhimurium, Serratia marcescens, and Staphylococcus aureus.* These strains are common contaminants of the environment in Egypt, some of which are involved in human and animal diseases or frequently reported from contaminated soil, water, and food substances. In addition, the antibacterial activity against clinical isolates of microorganisms from Assiut University Hospital was also investigated. Two different microorganisms were evaluated: *Staphylococcus aureus* and *Escherichia coli*, which are gram-positive and gram-negative resistant bacterial strains.

To inoculate for bioassay, bacterial strains were individually cultured for 48 h in universal tubes containing 15 mL nutrient broth medium. A bioassay was performed in 10 cm sterile plastic Petri plates in which microbial suspension (1 mL/plate) and 20 ml nutrient agar medium (20 mL/plate) were poured. After solidification of the media, 5 mm diameter cavities were cut in the solidified agar (3 cavities/plate) using a sterile corn borer. AgNPs at different concentrations were pipetted into the cavities (50 µL/cavity). Cultures were then incubated at 28 ± 0.5 °C for 48 h. Results were read as the diameter (in mm) of the inhibition zone around cavities (Kwon-Chung and Bennett 1992)^69^.

To determine the minimum inhibitory concentrations (MICs), AgNPs giving positive results, in terms of higher antibacterial activity, were diluted with DMSO to prepare a series of descending concentrations. Diluted AgNPs were similarly assayed as mentioned before, and the lowest concentration below which no activity was recorded as the MIC.

### Silver NPs quantification in bacterial cells

The quantitative determination of AgNPs after internalization into bacterial cells was determined using ICP-OES. Bacterial strains, *Escherichia coli* and *Staphylococcus aureus*, were grown and then incubated with different concentrations of formulated AgNPs for 24 h. Further, bacterial suspension was filtered for 5 min at 5000 rpm, and pellets were then digested with 3 mL of acidic mixture (nitric acid: perchloric acid; 3:1v/v), and then heated for 5 min at 50–70 °C. Samples were then diluted with deionized water to 25 mL and filtered using Whatman filter paper, and then analyzed with ICP-OES for quantitative determination of AgNPs inside bacterial cells.

### Morphological observation of bacteria after AgNPs internalization

The morphological examination of bacteria was examined after bacterial growth and incubation with different concentrations of the formulated AgNPs as those used in the MIC experiment. *Escherichia coli* and *Staphylococcus aureus* were chosen as a gram-negative and gram-positive bacteria, as they showed the highest inhibition zones for the following AgNPs concentrations (500, 42.62, 85.25, and 85.25 µM/mL for AgNPs-PEG, AgNPs-EC, AgNPs-MC, and AgNPs-HPMC, respectively). The broth dilution method was used in which AgNPs formulations were diluted with a broth and then mixed with bacterial cells. Briefly, different volumes of AgNPs formulations (0.125–2 ml) were added to sterile test tubes, followed by dilutions with different volumes of nutrient broth (2.0–3.875 mL) to obtain concentrations similar to those used in the MIC experiment (500–10.65 µM/mL). Then, each of the prepared AgNPs concentration was inoculated with 100 µl of bacterial broth and incubated for 24 h. After exposure of each bacterial species to different concentrations of the formulated AgNPs, the bacterial suspension was centrifuged for 10 min at 3000 rpm. Pellets obtained were washed with PBS (4x, each for 30 min), then stained with osmic acid (0.5 ml for 3 h, room temp, in dark conditions) and then rewashed with PBS (3x). Samples were then fixed in 2.0% agar, and further samples from agar were dehydrated using gradient concentrations of ethanol (70 for 30 min, 90 for 30 min, and 100% for 30 min), followed by 100% ethanol overnight followed by acetone for 30 min. Afterward, the embedding process was performed in a mold; subsequently, this medium was solidified to make a block for cutting thin sections of tissue for microscopic observation. A gradient embedding solution of approximately 0.5 mL (1:1v/v; ebon: acetone; 2:1 and 100% ebon) was added to each sample for 45 min, 45 min, and 1 h, respectively. Finally, samples were added into a mold (0.3 × 0.5 mL) and left to solidify for 48 h at 70 °C. Slices were cut using an ultra-section device followed by addition into gold grids for TEM observation.

### Statistical analysis

Statistical analysis was performed using one-way analysis of variance (ANOVA). Minitab 16 Statistical Software with Tukey's multiple evaluations was used to compare the preparations with each other. Statistically significant variances were approved when *p* ≤ 0.05. All values are expressed as mean ± standard deviation^54,70,71^.

## Conclusion

Efficient stable non-aggregated AgNPs were prepared using cellulosic polymers within an acceptable yield in a simple procedure. These cellulosic polymers could also stabilize the AgNPs formulations via efficient coating, as confirmed by UV–VIS and FT-IR transmission spectra results. The produced NPs had a geometrical particle size and zeta potential ranging from 88.4 ± 0.1 to 163 ± 0.09 nm & − 11.3 ± 0.5 to − 33.5 ± 0.9 mV, respectively. In addition, they showed a higher physical stability in terms of size and charge when stored at 25.0 ± 0.5 °C and 4.0 ± 0.5 °C for 3 months. A continuous release pattern was found regarding the release of silver cations from AgNPs formulations, with much more prolonged release being recorded with AgNPs-EC and AgNPs-HPMC, 48 and 36 h, respectively. AgNPs formulated using EC and HPMC showed a superior antioxidant activity and a significant (*p* ≤ 0.05; ANOVA/Tukey) antibacterial activity against the tested species of bacteria compared to AgNPs stabilized with MC, PEG, and those prepared without cellulosic stabilizer AgNPs-CIT. Furthermore, AgNPs stabilized with either EC or HPMC showed a more pronounced antibacterial effect on Gram-negative bacterial species such as *Pseudomonas aeruginosa, Salmonella typhimurium*, and *Serratia marcescens* compared with the tested Gram-positive bacterial species at a lower concentration of 21.31 µM/mL. Regarding the MIC, it was noticed that AgNPs stabilized via EC had lower MIC values compared to AgNPs stabilized with HPMC against the investigated Gram-positive and Gram-negative bacteria. In addition, cellulosic AgNPs showed superior antibacterial activity against multidrug-resistant clinical strains of *Escherichia coli* and *Staphylococcus aureus* presented at lower MIC values. Quantitative determination of AgNPs inside bacterial cells using ICP-OES revealed that a significant amount of AgNPs-HPMC and AgNPs-EC were embedded inside both *Escherichia coli* and *Staphylococcus aureus,* which supports the antibacterial studies compared with AgNPs-PEG and AgNPs-MC. In addition, morphological observation of both *Escherichia coli* and *Staphylococcus aureus* bacterial cells after AgNPs internalization using TEM showed higher amounts of cellulosic stabilized AgNPs found inside *Escherichia coli* cell interior compared with *Staphylococcus aureus*. Finally, it could be concluded that cellulosic polymers could be promising reducing and stabilizing agents for effective AgNPs formulation with an antioxidant activity and enhanced antibacterial action.

## Supplementary Information

Supplementary Information.
