# Supplementary material for: Different cellulosic polymers for synthesizing silver nanoparticles with antioxidant and antibacterial activities
Source: Sci Rep. 2021 Jan 8;11:84. doi: 10.1038/s41598-020-79834-6 (PMC7794214; doi:10.1038/s41598-020-79834-6)
Supplement: Supplementary file 1 — Supplementary Information. [file 41598_2020_79834_MOESM1_ESM.docx]

**Different cellulosic polymers for synthesizing silver nanoparticles with antioxidant and antibacterial activities**

Ahmed A. H. Abdellatif^1,2,*^, Hamad Al-Turki^1^, Hesham M. Tawfeek^3^

^1^ Department of Pharmaceutics, College of Pharmacy, Qassim University, Buraydah, 51452, Kingdom of Saudi Arabia

^2^ Department of Pharmaceutics and Industrial Pharmacy, Faculty of Pharmacy, Al-Azhar University, Assiut, 71524, Egypt

^3^ Department of Industrial Pharmacy, Faculty of Pharmacy, Assiut University, 71526, Assiut, Egypt

**Correspondence to:**

Dr. Ahmed A. H. Abdellatif

Associate Professor of Pharmaceutics:

- 1. College of Pharmacy, Qassim University, Buraydah, 51452, Kingdom of Saudi Arabia
  2. Faculty of Pharmacy, Al Azhar University, Assiut, 71524, Egypt

E-mail address:

[a.abdellatif@qu.edu.sa](mailto:a.abdellatif@qu.edu.sa)

[ahmed.a.h.abdellatif@azhar.edu.eg](mailto:ahmed.a.h.abdellatif@azhar.edu.eg)

Dr. Hesham M. Tawfeek

Associate Professor of Industrial Pharmacy, Department of Industrial Pharmacy, Faculty of Pharmacy, Assiut University, 71526, Assiut, Egypt.

E-mail address:

[heshamtawfeek@aun.edu.eg](mailto:heshamtawfeek@aun.edu.eg)

| 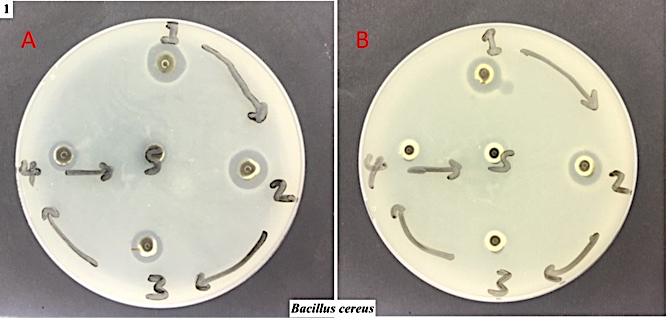 | 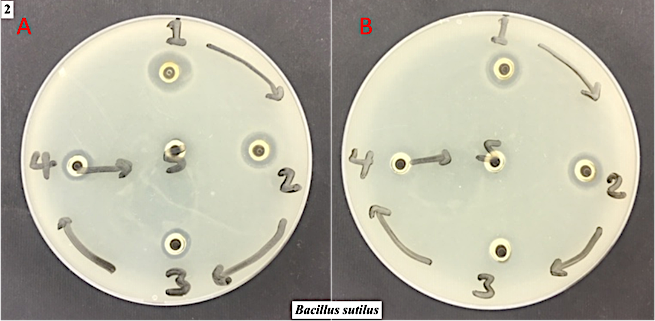 |
| --- | --- |
| 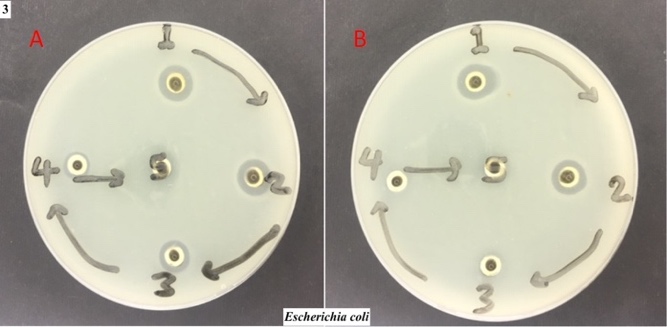 | 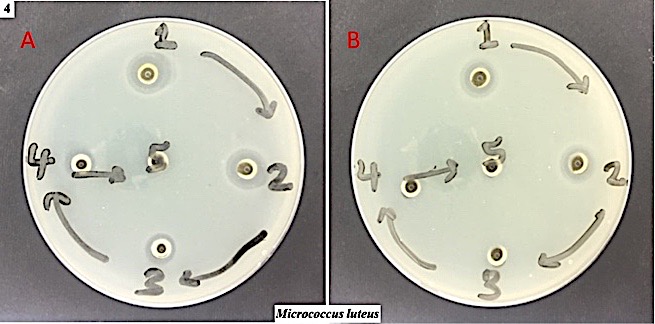 |
| 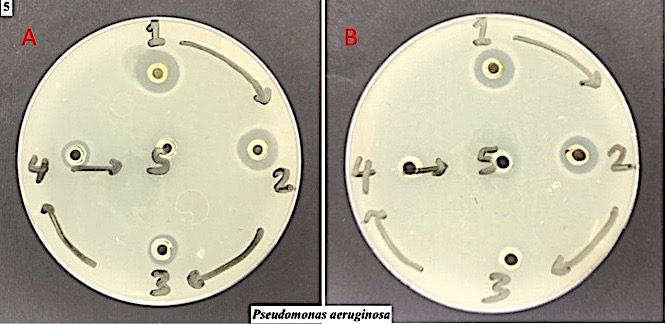 | 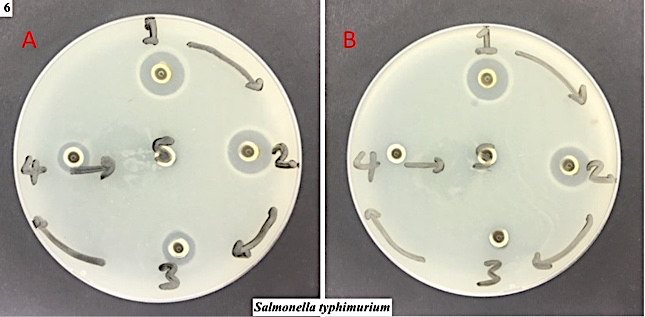 |
| 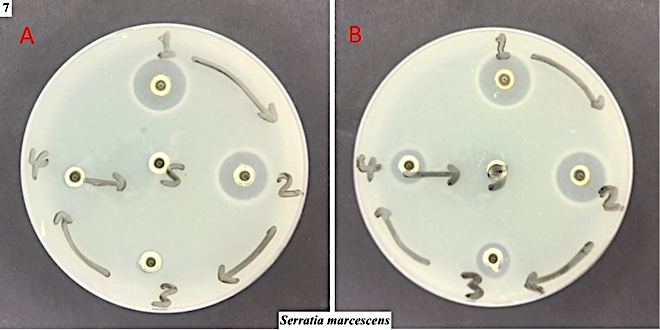 | 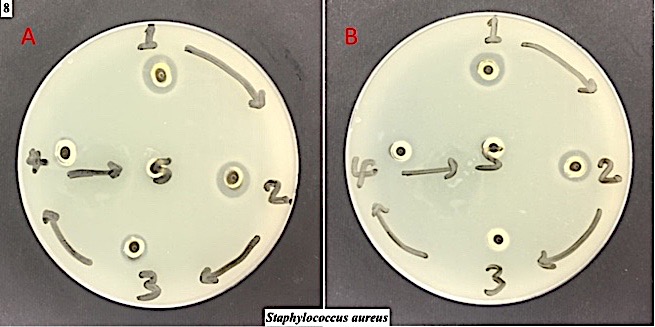 |

**S1.** Antibacterial activity expressed as the inhibition zone (mm) of AgNPs reduced with (A) ethyl cellulose and (B) HMPC. 1, 2, 3, 4, and 5 are the wells (cavities) containing different concentrations of AgNPs 341, 170.5, 85.25, 42.62, and 21.31 µM/mL, respectively.

.
